# Comprehensive High-Sensitivity Mutation Profiling in MPNs: Diagnostic and Prognostic Implications

**DOI:** 10.3390/cancers18162632

**Published:** 2026-08-14

**Authors:** Namsoo Kim, Yehyun Kang, Hye Won Kook, Haerim Chung, Ji Eun Jang, Seung-Tae Lee, Jaewoo Song, Jin Seok Kim, Jong Rak Choi, June-Won Cheong, Saeam Shin

**Affiliations:** 1 Department of Laboratory Medicine, Severance Hospital, Yonsei University College of Medicine, Seoul 03722, Republic of Korea; 2 Aerospace Medical Center, Republic of Korea Air Force, Cheongju 28187, Republic of Korea; 3 Genome Research Center, GC Genome, Yongin 16924, Republic of Korea; 4 Division of Hematology, Department of Internal Medicine, Severance Hospital, Yonsei University College of Medicine, Seoul 03722, Republic of Korea; 5 Dxome Co., Ltd., Seongnam-si 13209, Republic of Korea; 6 Blood Cancer Research Institute, Yonsei University College of Medicine, Seoul 03722, Republic of Korea

**Keywords:** myeloproliferative neoplasms, *JAK2*, *CALR*, *MPL*, variant allele frequency

## Abstract

**Background/Objectives:** Myeloproliferative neoplasms (MPNs) are clonal hematopoietic stem cell disorders driven by somatic mutations, most commonly those in *JAK2*, *CALR*, and *MPL*. While conventional molecular testing often focuses on these canonical mutations, emerging data suggest that additional mutations and low-variant-allele-frequency (VAF) subclones may contribute to disease progression and prognosis. **Methods:** We applied ultra-deep targeted sequencing with an error-correction algorithm to analyze bone marrow and peripheral blood samples from 134 patients with essential thrombocythemia (ET), polycythemia vera (PV), primary myelofibrosis (PMF), secondary myelofibrosis (MF), or post-MPN acute leukemia. Targeted sequencing panels were used to detect disease-associated and clonal driver mutations. Matched germline controls were not systematically available. **Results:** Low-VAF variants (<5%) were identified in 4/16 (25%) *CALR*, 3/7 (43%) *MPL*, and 9/11 (82%) *TP53* mutations. In contrast, all *TP53* mutations identified at leukemic transformation had VAFs > 5%, suggesting clonal expansion during progression. Paired sequencing at initial MPN diagnosis and leukemic transformation was available for three patients. *JAK2* p.V617F VAFs differed according to treatment timing (Kruskal–Wallis test, *p* = 0.0138), with lower VAFs observed in untreated patients; however, this association may be influenced by treatment-selection confounding. Mutations in *ASXL1* and *SRSF2* were occasionally observed in ET and secondary MF, although their prognostic significance remains uncertain. Progression analyses were exploratory because of the limited number of events and longitudinally sampled patients, and formal survival or prognostic modeling was not performed. **Conclusions:** Ultra-deep error-corrected sequencing enables sensitive detection of low-VAF mutations in MPNs, including subclonal events potentially missed by less sensitive assays. These findings demonstrate the potential utility of high-sensitivity panel-based sequencing for molecular characterization of MPN, while larger longitudinal studies are required to establish the prognostic and clinical significance of these findings.

## Simple Summary

This study evaluated ultra-deep error-corrected next-generation sequencing (NGS) in 134 patients with myeloproliferative neoplasms (MPNs) to determine its diagnostic and prognostic value. Ultra-deep sequencing detected many low-variant-allele-frequency (VAF < 5%) mutations that conventional methods could miss, including 25% of *CALR*, 43% of *MPL*, and 82% of *TP53* mutations. In contrast, most *JAK2* mutations had higher VAFs and were reliably detected by standard testing. During leukemic transformation, all TP53 mutations showed VAFs > 5%, suggesting clonal expansion during disease progression. Lower *JAK2* p.V617F VAFs were associated with patients who remained untreated, indicating potential clinical relevance for treatment decisions. Additional mutations such as *ASXL1* and *SRSF2* were observed in patients with disease progression. Overall, the findings suggest that panel-based ultra-deep NGS improves mutation detection, enhances diagnostic sensitivity, and may provide valuable prognostic information for MPN management, although larger prospective studies are needed to confirm these results.

## 1. Introduction

Myeloproliferative neoplasms (MPNs) are a group of clonal hematopoietic stem cell disorders characterized by sustained proliferation of myeloid lineages [1]. According to the 5th edition of the WHO classification, non-*BCR*::*ABL1* MPNs encompass polycythemia vera (PV), essential thrombocythemia (ET), and primary myelofibrosis (PMF), which are molecularly and clinically distinct from chronic myeloid leukemia [1]. While these entities share overlapping clinical features, they differ in prognosis and risk of progression to fibrotic or leukemic phases [2,3]. Molecular testing has become essential for diagnosis and classification, particularly with identification of disease driver mutations in *JAK2*, *CALR*, and *MPL* [4]. Beyond initial diagnosis, molecular profiling also aids in distinguishing cases at risk of progression, which can have significant therapeutic implications [5].

To improve diagnostic precision and prognostication, next-generation sequencing (NGS) panels have been widely implemented to detect both disease driver mutations and additional clonal driver mutations in genes such as *SRSF2*, *RUNX1*, *TP53*, *ASXL1*, *IDH1*, and *IDH2* [3,4]. These mutations are associated with clonal complexity and inferior clinical outcomes [6]. In routine clinical practice, highly sensitive allele-specific or quantitative PCR assays can detect predefined canonical driver mutations, particularly JAK2 p.V617F, at very low variant allele frequencies (VAFs) [7]. However, such assays are inherently restricted to predefined targets, whereas conventional targeted NGS enables broader mutational profiling but may have limited sensitivity for small mutant clones. Thus, an approach that combines broad genomic coverage with sensitive low-VAF detection could provide information complementary to highly sensitive single-target assays.

Importantly, the detection of a low-VAF mutation does not necessarily establish its disease-specific significance. Although low-level canonical *JAK2*, *CALR*, or *MPL* driver mutations may support an MPN diagnosis in the appropriate clinicopathologic context, mutations in other myeloid-associated genes may also represent clonal hematopoiesis and can occur in individuals without overt myeloid neoplasms. Consequently, the biological significance of very-low-VAF variants requires cautious interpretation, and reliable detection near the lower limit of an assay requires adequate analytical validation. Although technological advances now enable increasingly sensitive mutation detection, there is no consensus regarding the optimal sequencing depth or the clinical value of detecting very small mutant clones in MPNs [8]. Most existing data focus on dominant mutations, and few studies have explored the prevalence or clinical significance of low-VAF mutations across MPN subtypes [9]. Therefore, the prevalence and clinical implications of these small clones, particularly in relation to clonal evolution and disease progression, remain incompletely understood.

In this study, we applied PiSeq, an ultra-deep targeted sequencing method originally developed for detecting very-low-VAF mutations in circulating tumor DNA [10]. While PiSeq has primarily been used in liquid biopsy applications, it has recently shown utility in somatic mutation detection from unconventional sources such as vaginal swab DNA [11,12]. By adopting this high-sensitivity platform in a large cohort of MPN patients, we aimed to (i) comprehensively profile both disease driver and clonal driver mutations, including low-VAF variants, across MPN subtypes; (ii) identify molecular signatures suggestive of progression; and (iii) propose an optimized approach for applying NGS in the routine molecular workup of MPNs based on the clinical utility of ultra-deep sequencing.

## 2. Materials and Methods

### 2.1. Study Population

From January 2018 to April 2025, patients diagnosed with MPNs according to the 5th edition of the WHO classification and subjected to sequencing of bone marrow or peripheral blood samples were retrospectively enrolled in this study. For the purposes of this study, “initial” samples were defined as specimens obtained at the time of initial MPN diagnosis, whereas “follow-up” samples were defined as specimens obtained during subsequent follow-up while patients were receiving MPN-directed treatment. Secondary myelofibrosis was defined as myelofibrotic transformation arising from pre-existing PV or ET. Post-MPN leukemia was defined as leukemia diagnosed in patients with a previously established diagnosis of MPN (Supplementary Figure S1).

The study was approved by the Institutional Review Board (IRB) of Severance Hospital, Seoul, Republic of Korea (IRB No. 4-2025-0364), on 21 May 2025. The requirement for informed consent was waived due to the retrospective design and the use of anonymized data.

### 2.2. Targeted Sequencing

Genomic DNA was extracted from bone marrow or peripheral blood samples using a QIAamp DSP DNA Blood Mini Kit (Qiagen, Hilden, Germany). At the time of MPN diagnosis, patients underwent sequencing using either a 531- or 30-gene targeted panel (Dxome Co., Ltd., Seongnam-si, Republic of Korea). A custom hybrid capture panel (Dxome Co., Ltd.) was used for target enrichment, and paired-end sequencing (2 × 150 bp) was performed on a Novaseq 6000 (Illumina, San Diego, CA, USA). Base call (BCL) files generated by the sequencer were demultiplexed and converted to FASTQ files using bcl2fastq (Illumina). The bioinformatic pipelines for data analysis include demultiplexing, alignment of the reads to the reference genome GRCh37/hg19 with a Burrows–Wheeler Aligner (BWA-mem; v0.7.12), removing duplicates, base quality recalibration, and variant calling. To better detect low-frequency variants and minimize sequencing noise or PCR artifacts, an in-house positional indexing algorithm (PiSeq; Dxome) was used in additional variant calling steps [10]. Identified variants were annotated using the DxSeq platform version 2.2.5 (Dxome) with reference to publicly available databases. Copy number variants were identified according to previously described methods [13]. Detailed available analytical and bioinformatic performance metrics, including sequencing depth, mapping rate, on-target rate, and PiSeq-specific coverage metrics, are summarized in Supplementary Table S1.

### 2.3. Data Analysis, Variant Classification, and Interpretation

For this study, results were assessed based on 30 genes (Supplementary Table S2) common to both the 531-gene and 30-gene targeted panels. Although sequencing was performed using either the 531-gene or 30-gene targeted panel, the present study restricted comparative mutation analyses to the 30 genes shared by both panels to ensure a consistent gene set across patients. Variant classification was performed according to established guidelines from the Association for Molecular Pathology, American Society of Clinical Oncology, and College of American Pathologists [14]. Only Tier 1 or Tier 2 variants were included in the final analysis, and all included variants were manually reviewed using Integrative Genomics Viewer (Broad Institute, Cambridge, MA, USA) [15]. Transcript selection for variant annotation was based on MANE Select transcripts.

Matched germline controls were not systematically available. When a germline origin was clinically suspected, additional sequencing using skin-derived DNA was performed when available to distinguish germline from somatic variants. Low-VAF variants were manually reviewed but were not systematically confirmed using an independent orthogonal method. Gene fusion analysis was not performed in the present study.

For patients with multiple qualifying variants, all Tier 1 or Tier 2 variants meeting the study criteria were retained for analysis; multiple qualifying variants were identified in 67 patients. Three patients who subsequently developed post-MPN leukemia had paired sequencing samples obtained at initial MPN diagnosis and at leukemic transformation, which were retained for longitudinal analyses.

### 2.4. Statistical Analysis and Visualization

Statistical analysis and visualization were performed using R version 4.5.0 (R Core Team, Vienna, Austria, 2025) within the RStudio environment version 2024.04 (Rstudio Team, Boston, MA, USA, 2024). For continuous variables, the Mann–Whitney U test and Kruskal–Wallis test were used, while Fisher’s exact test followed by Benjamini–Hochberg false discovery rate correction and the χ^2^ test were applied for categorical variables. A *p*-value less than 0.05 was considered statistically significant. Oncoplots were generated using the maftools package in R [16].

### 2.5. Clinical Variables

Clinical variables including age, sex, initial hemoglobin or platelets, treatment choice, and prognosis were retrospectively obtained through review of electronic medical records. For the treatment-timing analysis, sequencing was performed using diagnostic samples obtained before the initiation of MPN-directed therapy. Patients were subsequently classified into three groups according to the interval between MPN diagnosis and treatment initiation: the immediate-treatment group, in which treatment was initiated at the time of diagnosis; the delayed-treatment group, in which treatment was initiated more than 1 month after diagnosis; and the untreated group, in which MPN-directed treatment was not initiated during the follow-up period. Thus, the treatment-timing analysis evaluated the association between baseline molecular findings and subsequent treatment timing rather than changes in variant allele frequency following treatment.

Disease progression was defined as transformation to secondary myelofibrosis or post-MPN leukemia. Follow-up duration was calculated from the date of initial MPN diagnosis to the date of the last available clinical follow-up or disease progression, as applicable. Detailed information on thrombosis history, splenomegaly, and symptom burden was not consistently or systematically documented in the medical records and was therefore not included in the analysis.

Sequencing was performed using either bone marrow or peripheral blood specimens. The numbers of bone marrow and peripheral blood specimens included in the analysis were 117 and 17, respectively.

## 3. Results

A total of 134 patients were diagnosed with ET, PV, PMF, secondary MF, or post-MPN acute leukemia (Table 1). ET was the most common subtype, accounting for 67 patients (initial 52, follow-up 15), followed by PV (*n* = 19; initial 15, follow-up 4) and PMF (*n* = 23; initial 19, follow-up 4). The highest proportion of *JAK2* p.V617F positivity was observed in PV, where 18 of 19 patients (94.7%) tested positive. Medical treatment modalities were broadly categorized into hydroxyurea, anagrelide, danazol, and ruxolitinib. Most ET patients were treated with hydroxyurea, with a minority receiving anagrelide. All treated initial PV patients received phlebotomy, and 80.0% also received hydroxyurea. Among patients with PMF and secondary MF, a variety of treatments were used, with ruxolitinib being the most frequently administered.

**Figure 1:**
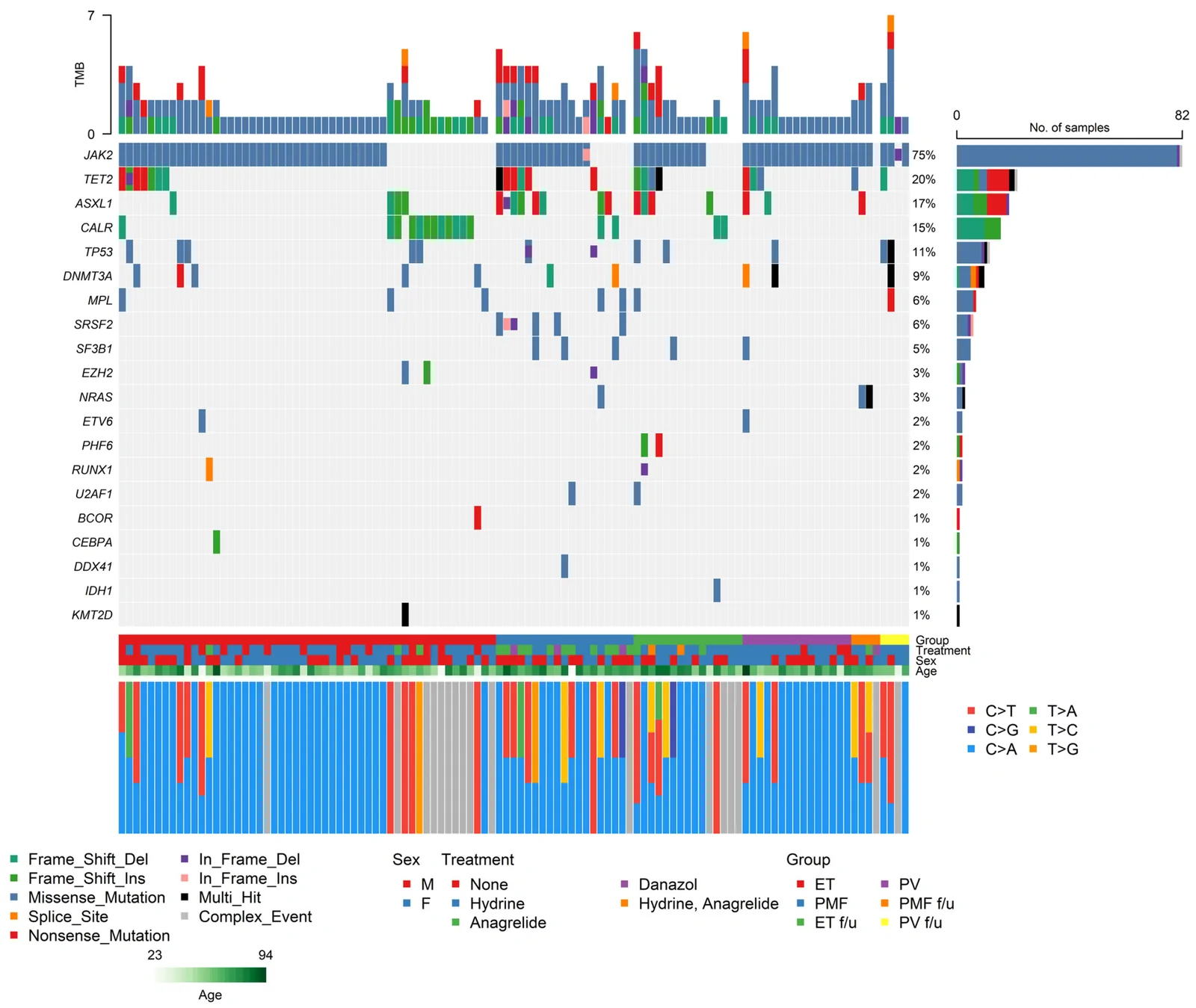
Oncoplot of mutation profiles in ET (*n* = 67), PV (*n* = 19), and PMF (*n* = 23) with exclusive and co-occurring mutations. Each column represents a single sample from a unique patient; repeat samples from the same patient were not included.

An oncoplot was generated for 109 patients with ET, PV, or PMF who had not progressed to secondary MF or post-MPN leukemia (Figure 1). After applying Benjamini–Hochberg false discovery rate correction, only the mutual exclusivity between *JAK2* and *CALR* remained statistically significant (*p* < 0.001), while all other pairwise associations did not remain statistically significant (Supplementary Data S1). To visually assess differences between the initial and follow-up time points, separate oncoplots were generated for each (Supplementary Figure S2). Although both groups were characterized by a high prevalence of *JAK2* mutations, *CALR* mutations were more frequent at initial diagnosis (14/86 vs. 2/23), and *TP53* mutations were more frequent at follow-up (8/86 vs. 4/23). However, neither difference reached statistical significance according to Fisher’s exact test. These patterns are likely influenced by differences in MPN subtype distribution between the two groups and should therefore be interpreted descriptively.

**Figure 2:**
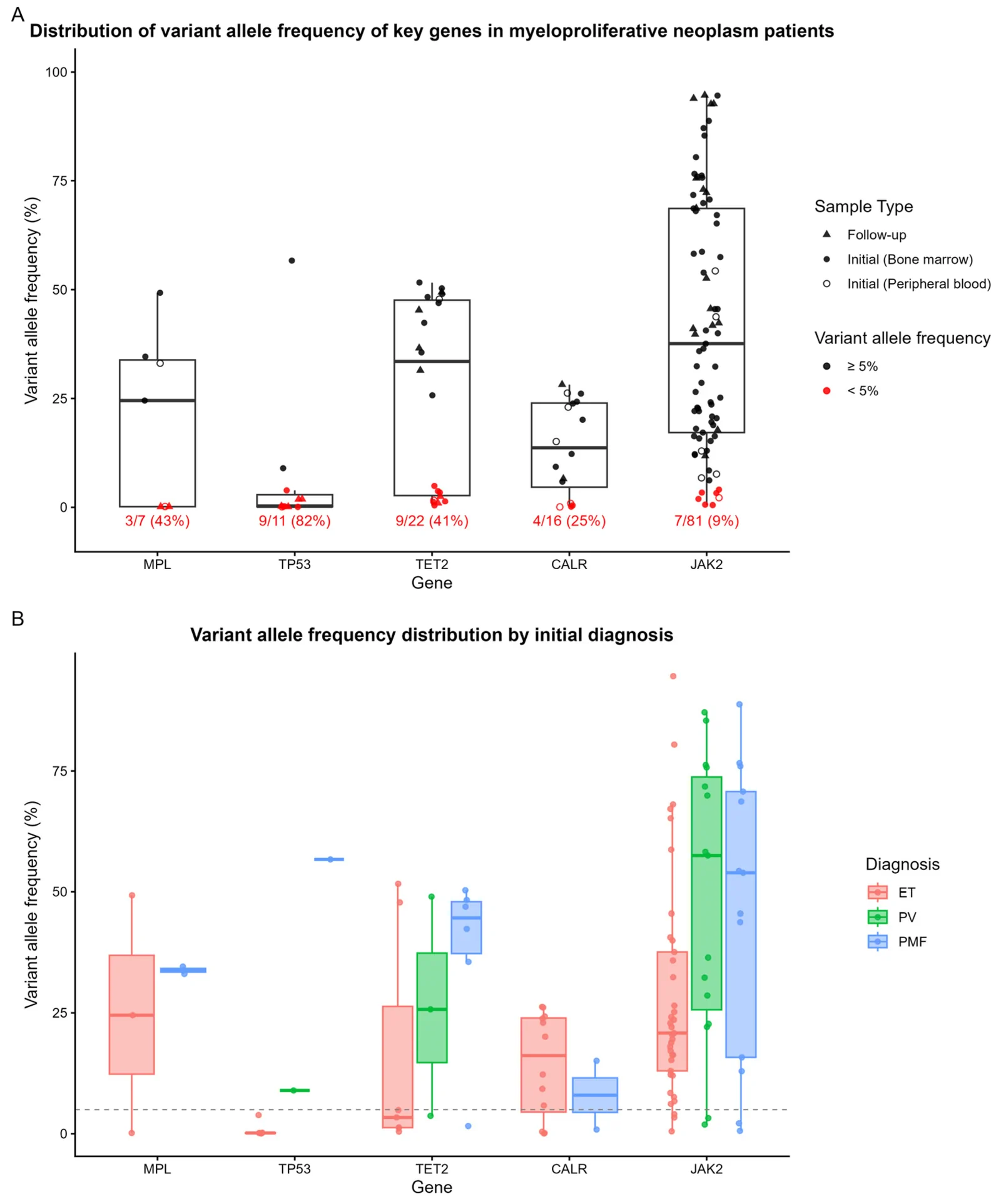
Distribution of variant allele frequencies of key genes in patients with myeloproliferative neoplasms. (**A**) Distribution of variant allele frequencies (VAFs) for *MPL* (*n* = 7), *TP53* (*n* = 11), *TET2* (*n* = 22), *CALR* (*n* = 16), and *JAK2* p.V617F (*n* = 81). The median VAFs (interquartile ranges) were 24.5% (0.1–33.8%) for *MPL*, 0.2% (0.1–2.9%) for *TP53*, 33.5% (2.7–47.6%) for *TET2*, 13.7% (4.6–23.9%) for *CALR*, and 37.6% (17.1–68.7%) for *JAK2* p.V617F. Variants with VAF < 5% are shown in red; the absolute number and proportion of low-VAF variants were 3/7 (43%) for *MPL*, 9/11 (82%) for *TP53*, 9/22 (41%) for *TET2*, 4/16 (25%) for *CALR*, and 7/81 (9%) for *JAK2* p.V617F. Point shapes indicate sample type and sampling time point: follow-up samples, initial bone marrow samples, and initial peripheral blood samples. (**B**) VAF distributions according to initial diagnosis of essential thrombocythemia (ET), polycythemia vera (PV), and primary myelofibrosis (PMF). Only samples obtained at initial diagnosis were included in this panel. The horizontal dashed line indicates a VAF threshold of 5%. VAFs were determined using PiSeq ultra-deep targeted next-generation sequencing.

Based on VAF from NGS, boxplots were constructed for key genes with recurrent mutations (Figure 2). Additionally, variants with VAF < 5%, which are often undetectable by conventional direct sequencing methods, were highlighted in red and counted separately. Among the most frequently mutated genes in MPN, *JAK2* mutations had a low proportion of VAF < 5% variants (9%), while *CALR* (25%), *MPL* (43%), *TET2* (41%), and especially *TP53* (82%) exhibited high proportions of low-VAF variants. When stratifying VAFs by gene and diagnostic category, variants with VAF < 5% were rarely observed in PV cases. In contrast, ET cases showed low-VAF (<5%) mutations across multiple genes, including *MPL*, *TP53*, *TET2*, *CALR*, and *JAK2*. In PMF, limited numbers of *CALR* and *JAK2* variants with VAF < 5% were also observed.

Among variants excluding canonical MPN driver mutations, a total of 101 alterations were identified in genes and regions associated with clonal hematopoiesis (CH) as defined by the WHO classification. Of these, 55 variants had a VAF ≥ 10%, whereas 46 had a VAF < 10%. At the patient level, 37 patients harbored at least one variant with VAF ≥ 10%, while 23 patients had only variants with VAF < 10%. The mean age of patients with VAF ≥ 10% variants was 69.2 years, compared with 63.3 years in those with only VAF < 10% variants.

To explore potential relationships between co-occurring mutations, we generated comparison plots of the VAFs of *JAK2* and other key genes in individual patients (Supplementary Figure S3). *JAK2* and *TET2* co-occurred in 15 patients; however, no significant correlation between their VAFs was observed. *TP53* co-occurred in five patients, with VAFs ranging from 0.078% to 56.687% (median, 0.200%); three of the five variants had VAFs < 5%. Furthermore, we examined the relationships between *JAK2* p.V617F VAF and initial hemoglobin and platelet levels in newly diagnosed patients (Supplementary Figure S4). Weak positive correlations were observed, but these did not reach statistical significance. Additionally, we examined whether patients with *JAK2* p.V617F positivity received medical therapy immediately, were treated in a delayed manner, or remained untreated, according to their individual VAF levels (Supplementary Figure S5). The untreated group exhibited lower VAFs of *JAK2* p.V617F, and the overall difference among the three groups was statistically significant (Kruskal–Wallis test, *p* = 0.0138).

Among our cohort, 10 patients met diagnostic criteria for MPN despite lacking detectable mutations in *JAK2*, *CALR*, and *MPL*. These included 6 cases of ET (3 at initial diagnosis and 3 during follow-up) and 4 cases of PMF (3 at initial diagnosis and 1 during follow-up). Of these 10 triple-negative cases, 1 ET patient remained untreated, whereas the remaining 5 ET patients received hydroxyurea. Among the 4 PMF patients, 3 were treated with ruxolitinib and 1 with danazol. Although a relatively high proportion of PMF patients received ruxolitinib and none experienced disease progression during the follow-up period, the small sample size limits definitive conclusions. NGS analysis identified no pathogenic variants in 5 of the 10 triple-negative patients, a proportion similar to that in patients with detectable mutations in the three major driver genes, among whom 50 out of 99 (50.5%) also showed no pathogenic variants. Among the remaining five triple-negative patients, *ASXL1* mutations were identified in three, two of whom exhibited low VAF. This proportion was higher than that observed in patients with mutations in the three major genes, among whom 4 out of 16 *ASXL1*-mutated cases showed low VAFs.

A total of 13 patients progressed from MPN to acute leukemia and underwent NGS analysis (Supplementary Table S3). These patients were diagnosed with acute leukemia at a mean of 10.2 years following their initial MPN diagnosis. Outcomes were generally poor: two patients achieved remission followed by engraftment, two were lost to follow-up, one failed to achieve remission, and eight died. The survival duration among deceased patients ranged from 4 days to 9 months. Notably, *TP53* mutations were detected in 6 of the 13 patients, 5 of whom died and 1 of whom was lost to follow-up. While *TP53* mutations in MPN were predominantly VAF < 5%, all *TP53* mutations identified during leukemic transformation had VAF exceeding 5%.

A total of 15 patients progressed from ET or PV to secondary MF (Supplementary Table S4). The average time to progression was 12.1 years. Unlike in cases that progressed to acute leukemia, most patients with secondary MF (*n* = 10) harbored no detectable mutations beyond *JAK2* or *CALR*. Importantly, none of these patients progressed to acute leukemia.

## 4. Discussion

This study investigates the application of NGS using the PiSeq method for detection of mutations in MPN, a field that has traditionally relied on PCR or conventional direct sequencing techniques in clinical settings [7]. Originally developed for the detection of extremely low VAFs in circulating tumor DNA, the PiSeq method has since demonstrated utility in other sample types—such as vaginal swab DNA—where detection of low-VAF mutations (e.g., below 5%) is necessary, depending on sequencing depth [12].

In the context of MPNs, molecular diagnostics have historically focused on three key genes: *JAK2*, *CALR*, and *MPL* [1]. By expanding the analysis to a broader gene panel consisting of 30 genes, our study enables a more comprehensive exploration of mutations associated not only with disease pathogenesis but also with progression to advanced disease states. Similarly, Wang et al. used a 42-gene NGS panel in patients with Ph-negative MPNs and demonstrated a broader mutational spectrum, including atypical variants coexisting with canonical *JAK2*, *CALR*, or *MPL* mutations [17].

These findings raise two important considerations regarding molecular testing in MPNs. First, molecular profiling beyond the canonical *JAK2*, *CALR*, and *MPL* drivers and *TP53* may benefit from inclusion of recurrently mutated genes involved in clonal evolution and disease progression, such as *ASXL1*, *TET2*, *SRSF2*, *SF3B1*, *U2AF1*, *EZH2*, *IDH1/2*, and *DNMT3A*. Broader profiling of these genes may provide a more comprehensive characterization of the molecular landscape of MPNs. Second, the analytical sensitivity of the sequencing method itself should be considered. Conventional NGS assays with a VAF detection threshold of approximately 1–2% may fail to detect very small clones or may classify them as sequencing noise. Therefore, when detection of low-VAF variants is clinically or biologically relevant, analytically validated high-sensitivity approaches incorporating error-correction strategies may provide additional information. Nevertheless, the clinical significance of very-low-VAF variants remains uncertain and requires validation in larger longitudinal studies.

Among the three critical diagnostic genes in MPNs, *JAK2*, *CALR*, and *MPL*, the *JAK2* p.V617F mutation is routinely detected by PCR in clinical practice. Since only a small proportion (<10%) of *JAK2*-mutated samples exhibited a VAF less than 5%, the risk of false-negative results for *JAK2* due to low VAF is considered minimal. In contrast, *CALR* and *MPL* mutations are often identified via direct sequencing methods, which can be less sensitive at low VAF levels. Our data showed that 42.9% of *MPL* mutations and 25.0% of *CALR* mutations had VAF < 5%, suggesting that reliance on conventional direct sequencing could lead to under-detection of these mutations.

Moreover, the distribution of mutations differed by MPN subtype. In PV, which is predominantly associated with *JAK2*, only two cases showed a VAF < 5%. However, ET cases demonstrated greater diversity, with mutations observed in *JAK2*, *CALR*, and *MPL*. Given that mutations in these genes are part of the major diagnostic criteria for ET, and that 37 of 52 patients (71.2%) with an initial ET diagnosis received treatment, the possibility of false-negative results due to insufficient sequencing sensitivity can impact treatment eligibility.

Genes known to be associated with leukemic transformation in PV include *SRSF2*, *RUNX1*, *TP53*, *ASXL1*, *IDH1*, and *IDH2* [3,18]. In ET, leukemic progression is linked to mutations in *TP53* and *RUNX1*, while in PMF, *TP53* is the key gene involved in leukemic transformation. In our study, among these genes, *TP53* was the most recurrently mutated. Notably, most *TP53* mutations (82%) had VAFs less than 5%, highlighting the clinical relevance of NGS methods such as PiSeq for detecting somatic mutations at low VAFs.

In addition, we evaluated variants occurring in genes associated with CH as defined by the WHO classification [1]. The mean age of patients harboring variants with VAF ≥ 10% was higher than that of patients with only VAF < 10% variants, which is consistent with the known age-related increase in CH. However, these findings suggest that the observed mutations cannot be solely attributed to low-level, age-related CH and may instead represent a mixture of CH-related and disease-associated subclonal variants.

Nevertheless, low-VAF variants in CH-associated genes, including *TP53*, *TET2*, and *DNMT3A*, may still represent background CH and should therefore be interpreted with caution. Notably, *TP53* and *TET2* mutations were relatively frequent in the low-VAF range, and previous studies have reported their association with disease progression and poor prognosis [19,20].

Due to the study design, it was not possible to clearly distinguish CH-related variants from disease-associated mutations. Therefore, these findings should be interpreted as hypothesis-generating, and further longitudinal studies are needed to clarify their biological and clinical significance.

Among 13 patients with MPNs who progressed to acute leukemia, 8 cases (3 ET and 5 PV) harbored mutations in the genes associated with leukemic transformation that corresponded with their initial MPN subtype. This high concordance suggests that NGS at the time of initial MPN diagnosis can help identify patients at higher risk of poor prognosis and inform early therapeutic decision-making.

In addition, among patients diagnosed with post-ET or post-PV myelofibrosis (MF), NGS analysis revealed a low frequency of leukemic transformation-related gene mutations, with only one *TP53* mutation identified among 16 cases of post-ET MF. Notably, previous studies have infrequently mentioned [21,22]. *SRSF2*, *ASXL1*, *IDH1*, or *IDH2* as leukemic transformation–associated genes in ET. However, in our cohort of four patients with acute leukemia who had a prior diagnosis of ET, one *SRSF2* and two *ASXL1* mutations were identified. Additionally, among eight patients with post-ET MF, one *ASXL1* and one *SRSF2* mutation were observed. While both *SRSF2* and *ASXL1* are reported at a frequency less than 10% in ET, their presence in these patients suggests a potential role in disease progression.

Among the eight patients with leukemic transformation who initially harbored the *JAK2* p.V617F mutation during the MPN phase, four were found to be negative for the mutation at the time of acute leukemia diagnosis. This loss of the driver mutation suggests a shift in clonal dominance during leukemic transformation, whereby a *JAK2*-wild-type subclone, previously minor or undetectable, may have expanded to become the predominant leukemic clone. Similar patterns have been reported in prior studies, where *JAK2*-mutant MPNs gave rise to *JAK2*-wild-type acute leukemia, supporting the concept of clonal evolution and parallel clonal trajectories in MPN progression [23]. These findings underscore the heterogeneity of leukemic transformation in MPNs and suggest that molecular profiling at the time of transformation is essential to capture such clonal shifts.

Currently, the prognostic significance of *ASXL1* mutations in ET remains unclear, with limited reports available beyond the observation that *ASXL1* is not prognostically significant in MF following ET [24]. For *SRSF2*, some studies have reported a significant correlation between *SRSF2* mutations and inferior overall survival in ET patients, but these findings remain insufficient to draw definitive conclusions [25,26]. Therefore, panel-based NGS approaches capable of detecting such mutations at low VAFs are essential for elucidating the prognostic implications of *SRSF2* and *ASXL1* mutations in ET.

Various analyses related to the VAFs of multiple genes showed that VAF itself did not show significant clinical relevance for the majority of mutations. This might be due to the lack of uniform biological effects of different mutations within the same gene [27]. In contrast, a focused analysis on *JAK2* p.V617F demonstrated meaningful findings. Specifically, we stratified patients with *JAK2* p.V617F mutations into three groups—immediate treatment, delayed treatment, and no treatment—and compared their VAFs. This analysis revealed a significant difference among the groups.

Traditionally, *JAK2* p.V617F testing via PCR or sequencing in clinical settings has provided only qualitative (positive/negative) results, without reporting VAF values. Furthermore, no clinical guidelines have been established regarding interpretation of *JAK2* p.V617F VAF in therapeutic decision-making [8]. However, our results showed that patients in the untreated group tended to have significantly lower *JAK2* p.V617F VAFs, and the intergroup comparison yielded significant differences. Given that recent clinical trials are investigating the association between *JAK2* p.V617F VAF and patient risk [28], our findings support *JAK2* VAF as a potential criterion for both therapeutic intervention and prognostic stratification.

Therefore, based on our findings, we propose a molecular genetic approach to the evaluation of MPN patients as follows. In clinical laboratories, various testing strategies are currently in use. Some centers rely solely on *JAK2* p.V617F PCR testing in accordance with older diagnostic criteria, while others focus on diagnostic efficiency and perform sequencing of only the three canonical genes—*JAK2*, *MPL*, and *CALR* [7]. However, in the present study, we identified multiple variants with VAF < 5% across all three genes, highlighting the potential for missed diagnoses when relying solely on conventional methods. Furthermore, we found that genes known to be associated with poor prognosis were frequently mutated in patients who had progressed, and our analysis suggested an association between *JAK2* p.V617F VAF and treatment decisions. Taken together, these findings support the need for panel-based, high-depth NGS that includes genes known to be clinically relevant, not only to enhance diagnostic sensitivity but also to provide prognostically and therapeutically relevant information.

There were several limitations to this study. First, this was a single-institution retrospective study with a relatively small sample size, particularly for patients who progressed to acute leukemia or secondary myelofibrosis, limiting the statistical power for evaluating disease progression and clinical outcomes. Second, although ultra-deep sequencing enabled the detection of variants at very low VAFs, orthogonal confirmation of low-VAF variants was not routinely performed, and matched germline samples were not systematically available. Therefore, very-low-VAF variants and variants with a potential germline origin should be interpreted cautiously. In addition, although available analytical performance metrics are provided, comprehensive analytical validation across the full range of low VAFs was limited by the retrospective nature of the study. Third, two targeted sequencing panels with different sequencing depths and analytical characteristics were used, and both bone marrow and peripheral blood specimens were included, introducing panel- and specimen-related heterogeneity. Fourth, the biological significance of low-VAF variants, particularly those involving genes associated with clonal hematopoiesis, cannot always be distinguished from disease-related clonal alterations and therefore requires cautious interpretation. Fifth, treatment was determined according to clinical practice rather than randomly assigned; consequently, the observed association between *JAK2* p.V617F VAF and treatment timing may be affected by treatment-selection confounding and should not be interpreted as evidence of a causal relationship. Sixth, only a small number of patients underwent sequencing at multiple disease time points. Accordingly, most comparisons were cross-sectional, and longitudinal clonal evolution cannot be inferred from these data. Seventh, the limited number of progression events precluded formal multivariable prognostic modeling, and the prognostic implications of the identified variants therefore remain exploratory. Finally, although our targeted sequencing approach enabled detection of DNA mutations and copy-number alterations across clinically relevant genes, it did not assess gene rearrangements or epigenetic alterations such as DNA methylation. Future prospective studies incorporating systematic orthogonal validation, matched germline controls, standardized sequencing and specimen types, longitudinal sampling, and sufficiently large cohorts for formal prognostic modeling will be needed to validate these findings and further refine molecular risk stratification in MPN.

## 5. Conclusions

In this study, we demonstrated the utility of NGS using the PiSeq to detect low-VAF variants in patients with myeloproliferative neoplasms. While *JAK2* mutations were largely detectable with conventional methods due to their typically high VAFs, a substantial proportion of *CALR*, *MPL*, and *TP53* mutations exhibited VAFs less than 5%, highlighting the potential for false-negative results when using limited or low-sensitivity assays. Importantly, low-VAF *TP53* mutations were prevalent even before leukemic transformation, whereas all TP53 mutations identified at transformation to acute leukemia exhibited VAFs greater than 5%, suggesting a potential clonal expansion preceding disease progression.

Our findings support the potential clinical utility of sensitive NGS assays in the diagnostic and prognostic evaluation of MPNs, particularly for detecting mutations associated with disease progression. Broader implementation of ultra-deep sequencing in selected patients might enhance risk stratification and inform early therapeutic decisions. Future studies should further validate these observations in prospective cohorts and clarify the optimal timing and indications for high-sensitivity sequencing in MPN management.

## Figures and Tables

**Table 1:** Baseline characteristics of patients with myeloproliferative neoplasm subtypes (total *n* = 134).

	Initial Diagnosis			Follow-Up			Secondary MF (*n* = 15)		Post-MPN Leukemia (*n* = 10)
	ET (*n* = 52)	PV (*n* = 15)	PMF (*n* = 19)	ET (*n* = 15)	PV (*n* = 4)	PMF (*n* = 4)	sMF from ET (*n* = 7)	sMF from PV (*n* = 8)	
Male sex, *n*(%)	25/52 (48.1%)	7/15 (46.7%)	13/19 (68.4%)	6/15 (40.0%)	1/4 (25.0%)	2/4 (50.0%)	0/7 (0.0%)	3/8 (37.5%)	7/10 (70.0%)
Age, years, median (IQR)	59 (52–70)	63 (57–73)	70 (65–75)	69 (60–79)	58 (52–60)	68 (66–69)	48 (48–59)	64 (57–69)	70 (67–79)
JAK2 V617F, *n*(%)	37/52 (71.2%)	15/15 (100.0%)	13/19 (68.4%)	10/15 (66.7%)	3/4 (75.0%)	3/4 (75.0%)	4/7 (57.1%)	8/8 (100.0%)	7/10 (70.0%)
JAK2 exon12/CALR/MPL, *n*(%)	13/52 (25.0%)	0/15 (0.0%)	3/19 (15.8%)	3/15 (30.0%)	2/4 (50.0%)	0/4 (0.0%)	3/7 (42.9%)	0/8 (0.0%)	1/10 (10.0%)
Treatment, *n*(%)	37/52 (71.2%)	12/15 (80.0%)	19/19 (100.0%)	15/15 (100.0%)	4/4 (100.0%)	4/4 (100.0%)	7/7 (100.0%)	7/8 (87.5%)	-
Hydroxyurea, *n*(%) among treated	34/37 (91.9%)	12/12 (100.0%)	5/19 (26.3%)	13/15 (86.7%)	4/4 (100.0%)	2/4 (50.0%)	3/7 (42.9%)	3/7 (42.9%)	-
Anagrelide, *n*(%) among treated	3/37 (8.1%)	0/12 (0.0%)	0/19 (0.0%)	4/15 (26.7%)	0/4 (0.0%)	0/4 (0.0%)	2/7 (28.6%)	0/7 (0.0%)	-
Danazol, *n*(%) among treated	0/37 (0.0%)	0/12 (0.0%)	3/19 (15.8%)	0/15 (0.0%)	0/4 (0.0%)	1/4 (25.0%)	0/7 (0.0%)	0/7 (0.0%)	-
Ruxolitinib, *n*(%) among treated	0/37 (0.0%)	0/12 (0.0%)	11/19 (57.9%)	0/15 (0.0%)	0/4 (0.0%)	1/4 (25.0%)	2/7 (28.6%)	4/7 (57.1%)	-

## Data Availability

The data supporting the findings of this study are available within the article and its Supplementary Materials. Other datasets used and/or analyzed during the current study are available from the corresponding author (S.S.) on reasonable request.

## Supplementary Materials

The following supporting information can be downloaded at https://www.mdpi.com/article/10.3390/cancers18162632/s1. Table S1: Sequencing quality control metrics according to targeted sequencing panel. Table S2: List of 30 genes included in MRD30 panel. Table S3: Demographics in patients with myeloproliferative neoplasm that progressed to acute leukemia. Table S4: Demographics in patients with myeloproliferative neoplasms that progressed to myelofibrosis. Figure S1: Sample flow diagram of study. Figure S2: Oncoplot of mutation profiles at (A) initial diagnosis and (B) follow up in myeloproliferative neoplasm patients. Figure S3: Comparison of *JAK2* with (A) *TP53* and (B) *TET2* mutations in primary diagnosis of essential thrombocythemia (ET), polycythemia vera (PV), and primary myelofibrosis (PMF). Figure S4: Comparison of (A) *JAK2* p.V617F variant allele frequency (%) with initial hemoglobin in PV and (B) *JAK2* p.V617F variant allele frequency (%) with initial platelet counts in ET. Spearman correlation coefficients and *p*-values are indicated in each panel. Figure S5: Comparison of *JAK2* p.V617F variant allele frequency (%) across therapy timepoints. Supplementary Data S1: Pairwise co-occurrence and mutual exclusivity analysis of selected genes in MPN cohort.

## Abbreviations

The following abbreviations are used in this manuscript:

MPN	Myeloproliferative neoplasm
PV	Polycythemia vera
ET	Essential Thrombocythemia
PMF	Primary myelofibrosis
NGS	Next-generation sequencing
VAF	Variant allele frequency
IRB	Institutional Review Board
