# Supplementary material for: Comprehensive High-Sensitivity Mutation Profiling in MPNs: Diagnostic and Prognostic Implications
Source: Cancers (Basel). 2026 Aug 14;18(16):2632. doi: 10.3390/cancers18162632 (PMC13510349; doi:10.3390/cancers18162632)
Supplement: Supplementary file 1 [file cancers-18-02632-s001.zip › Supplement_R1_.pdf]

Supplementary Figure S1. Sample flow diagram of Study

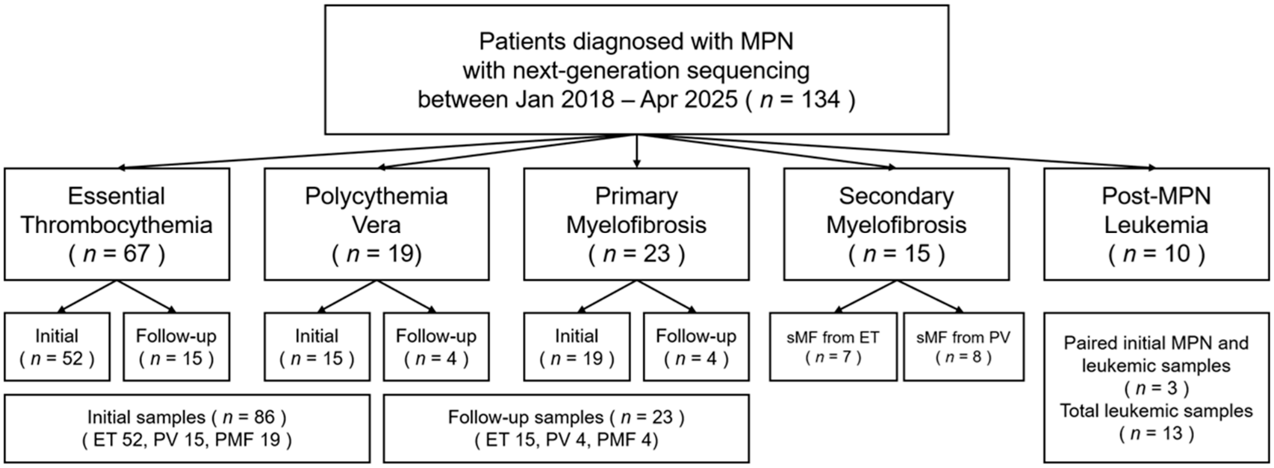

Supplementary Figure S2. Oncoplot of mutation profiles at (A) initial diagnosis and (B) follow up in myeloproliferative neoplasm patients

(A)

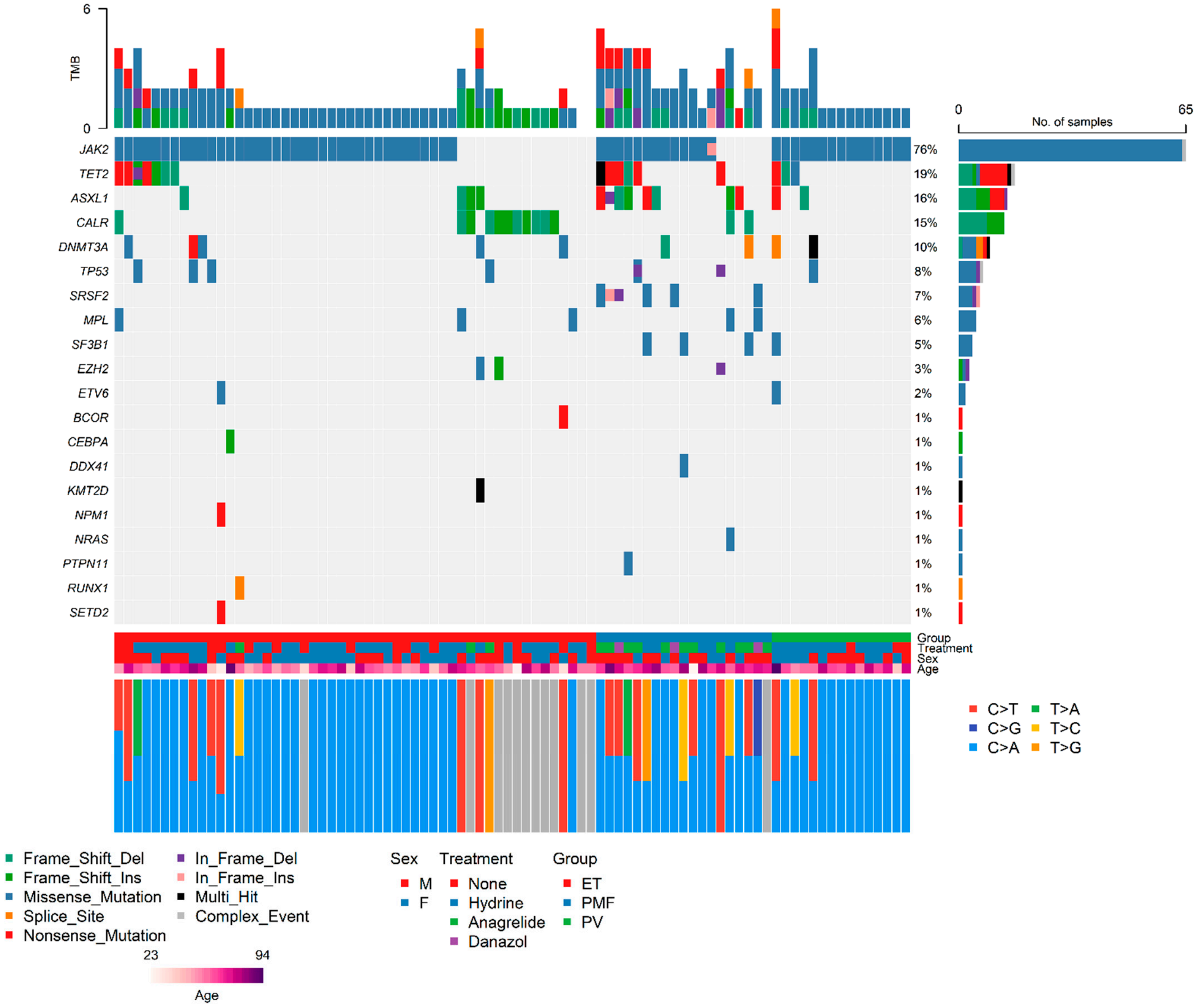

(B)

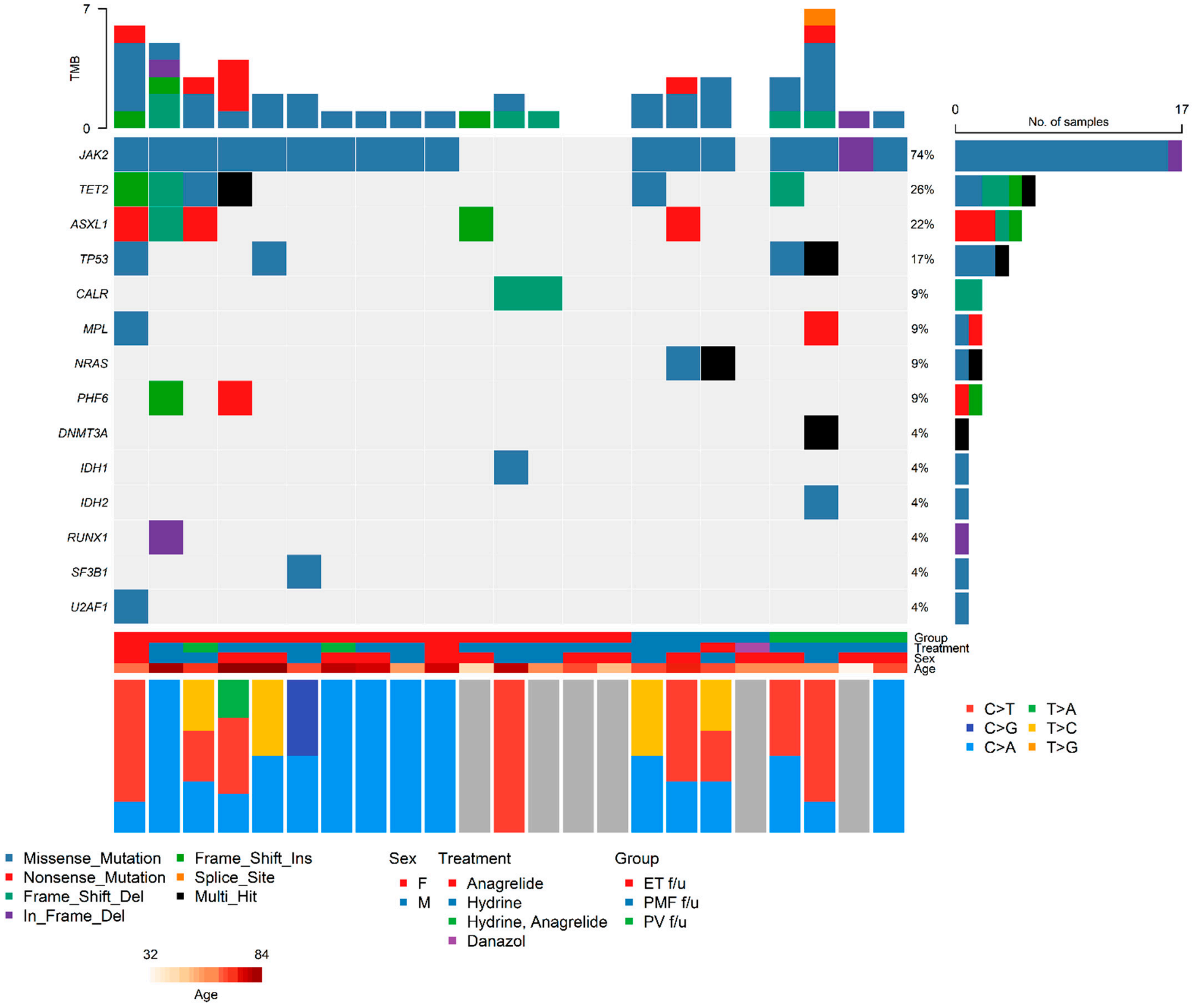

TMB
7
0
JAKS2
TET2
ASXL1
TP53
CALR
MPL
NRAS
PHF6
DNMT3A
IDH1
IDH2
RUNX1
SF3B1
U2AF1
74%
26%
22%
17%
9%
9%
9%
9%
4%
4%
4%
4%
4%
4%
0
No. of samples
17
Group
Treatment
Sex
Age
C>T
C>G
C>A
T>A
T>C
T>G
Missense_Mutation
Nonsense_Mutation
Frame_Shift_Del
In_Frame_Del
Frame_Shift_Ins
Splice_Site
Multi_Hit
Sex
F
M
Treatment
Anagrelide
Hydrine
Hydrine, Anagrelide
Danazol
Group
ET f/u
PMF f/u
PV f/u
32
84
Age

Supplementary Figure S3. Comparison of *JAK2* with (A) *TP53*, (B) *TET2* mutations in primary diagnosis of essential thrombocythemia (ET), polycythemia vera (PV), and primary myelofibrosis (PMF)

(A) (B)

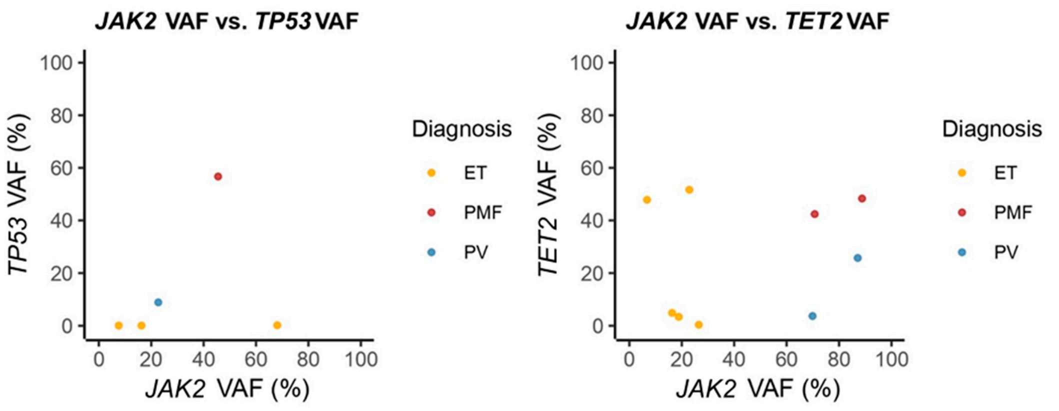

Supplementary Figure S4. Comparison of (A) *JAK2* p.V617F variant allele frequency (%) with initial hemoglobin in PV and (B) *JAK2* p.V617F variant allele frequency (%) with initial platelet counts in ET. Spearman correlation coefficients and p-values are indicated in each panel.

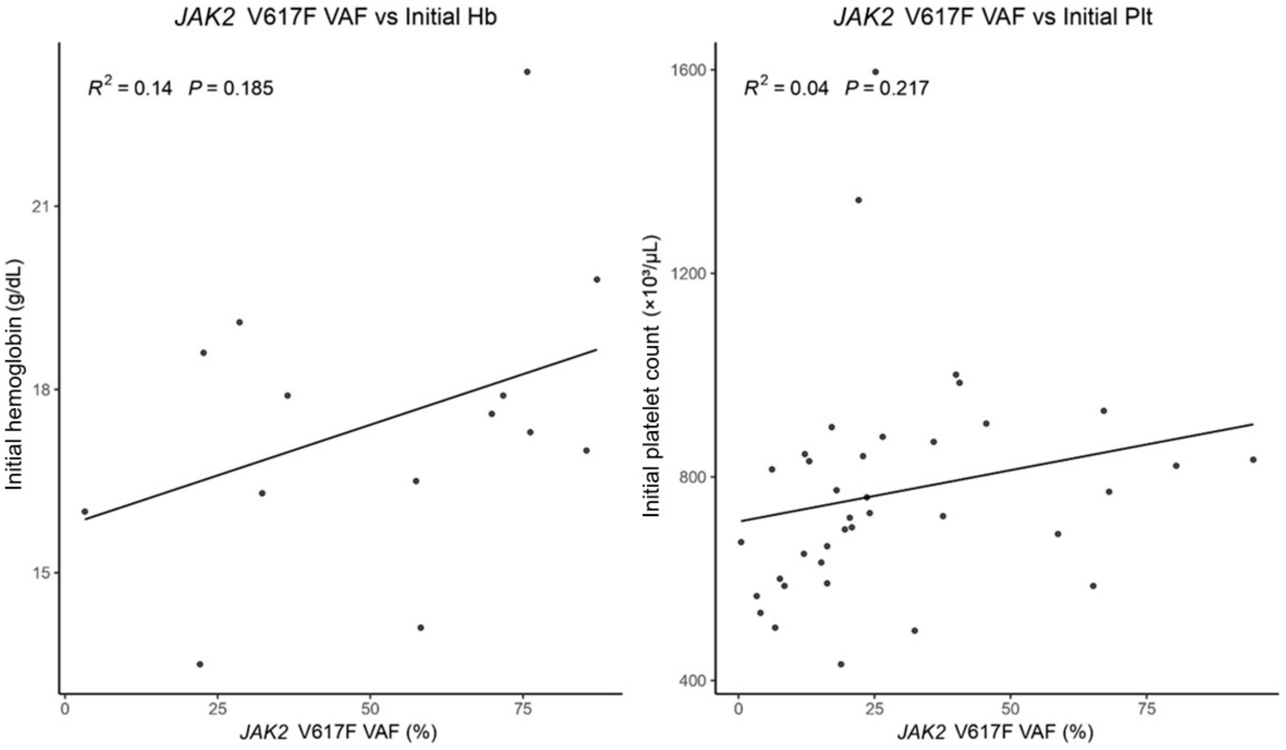

Supplementary Figure S5. Comparison of *JAK2* p.V617F variant allele frequency (%) across therapy timepoints.

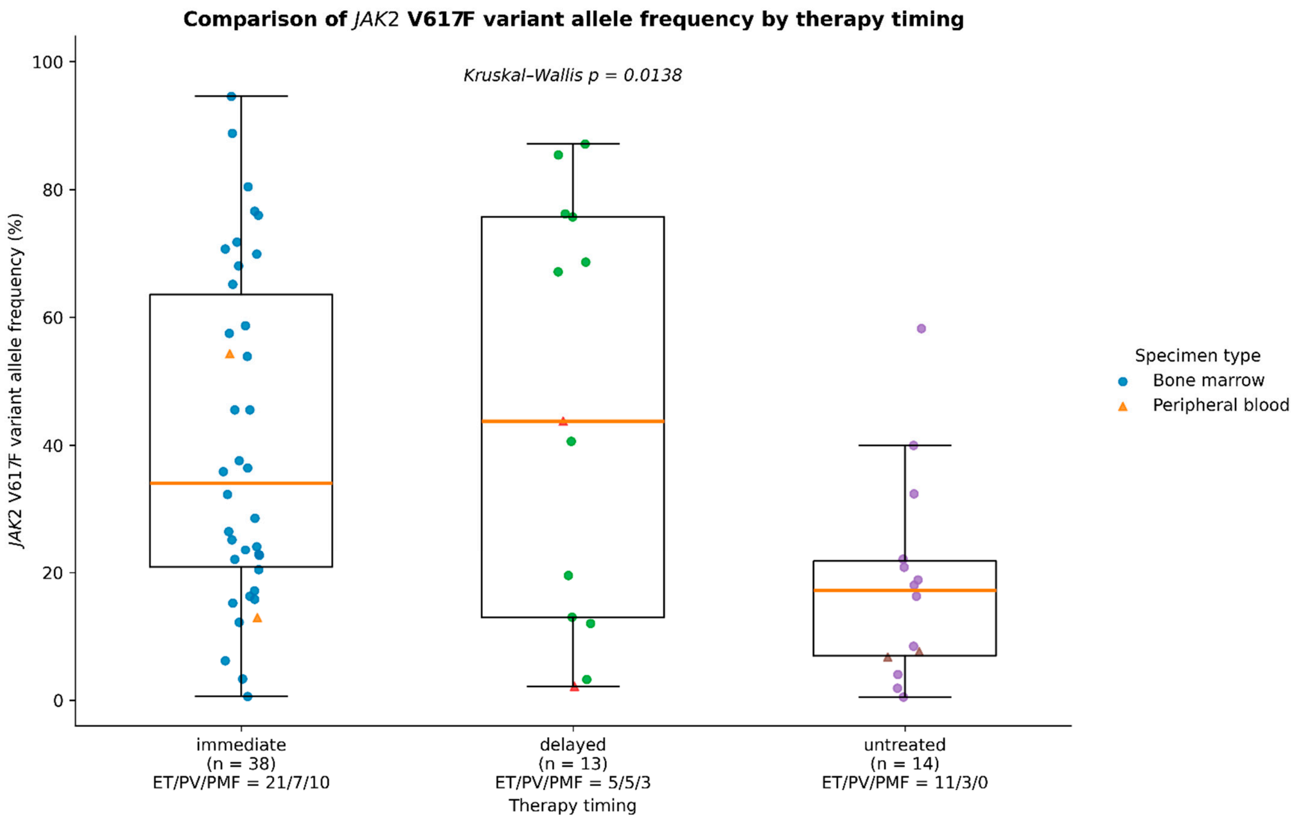

Supplementary Table S1. Sequencing quality control metrics according to targeted sequencing panel

QC metric	Hema500 (n=114), median (IQR)	MRD30 (n=9), median (IQR)
Average depth	2,862× (1,573–3,807)	50,367× (45,505–71,169)
Mapped bases	99.6% (99.5–99.7)	99.8% (99.7–99.9)
Mapped reads	100.0% (99.9–100.0)	100.0% (100.0–100.0)
Properly paired reads	99.3% (99.2–99.4)	99.3% (99.1–99.4)
Duplicate rate	19.6% (16.0–25.8)	81.0% (75.0–86.4)
Average quality	35.6 (34.4–35.9)	36.2 (36.2–36.3)
On-target rate	54.5% (52.5–60.2)	57.1% (49.7–61.3)
Total number of molecular barcodes	12,085,911 (7,332,046–15,128,605)	3,710,867 (3,625,555–3,893,226)
Duplex matched molecular barcodes	9.2% (7.1–11.5)	37.5% (37.4–46.0)
Median number of duplicates per molecular barcode	1.0 (1.0–1.0)	4.0 (4.0–6.0)

Supplementary Table S2. List of 30 genes included in MRD30 panel.

ASXL1	*BCOR*	*CALR*	*CEBPA*	*DDX41*	*DNMT3A*
ETV6	*EZH2*	*FLT3*	*IDH1*	*IDH2*	*JAK2*
KIT	*KMT2D*	*KRAS*	*MPL*	*NPM1*	*NRAS*
PHF6	*PTPN11*	*RAD21*	*RUNX1*	*SETD2*	*SF3B1*
SRSF2	*STAG2*	*TET2*	*TP53*	*U2AF1*	*WT1*

Supplementary Table S3. Demographics in patients with myeloproliferative neoplasm that progressed to acute leukemia

Patient No.	Sex	Age at MPN diagnosis	MPN	Age at leukemic transformation	Type	Time to leukemic transformation (years)	Molecular result at MPN diagnosis (VAF)*	Molecular result at leukemic transformation (VAF)*	Status after initial diagnosis
121	F	51	ET	70	Acute leukemia	19	Not available	*JAK2* c.1849G>T p.Val617Phe (1.05%)<br>*TP53* c.736A>G p.Met246Val (72.03%)<br>*TET2* c.483dup p.Asp162ArgfsTer9 (0.05%)<br>*RAD21* c.1032T>A p.Tyr344Ter (22.46%)	Follow-up Loss (1 month)
136	M	60	ET	78	Acute leukemia	18	Not available	*JAK2* c.1849G>T p.Val617Phe (41.7%)<br>*FLT3* c.1759_1800dup p.Asn587_Asp600dup (12.9%)<br>*U2AF1* c.101C>A p.Ser34Tyr (45.4%)	Follow-up Loss (9 months)
86	M	71	ET	75	Acute leukemia	4	*JAK2* V617F PCR positive	*TP53* c.818G>A p.Arg273His (26.7%)<br>*TP53* c.375G>A p.Thr125= (7.6%)<br>*TP53* whole gene deletion<br>*JAK2* c.1849G>T p.Val617Phe (36.5%)<br>*RUNX1* c.497G>A p.Arg166Gln (12.0%)<br>*SRSF2* c.284C>A p.Pro95His (36.0%)<br>*ASXL1* c.2790_2791delinsAA p.Trp930_Glu931delinsTer (37.1%)	Death (1 month)

87	F	62	ET	73	Acute leukemia	11	*JAK2* V617F PCR positive	*ASXL1* c.1773C>A p.Tyr591Ter (43.5%)<br>*RUNX1* c.967_967+1insCCGAA p.Ala324GlufsTer6 (42.4%)<br>*BCOR* c.4875-2A>T (14.6%)<br>*NRAS* c.35G>A p.Gly12Asp (12.4%)<br>*NRAS* c.37G>A p.Gly13Arg (29.6%)	Death (5 months)
88	M	54	PV	67	Acute leukemia	13	Not available	*JAK2* c.1849G>T p.Val617Phe (94.4%)<br>*RUNX1* partial gene deletion<br>*ASXL1* whole gene deletion<br>*TP53* whole gene deletion	Death (1 month)
137	F	43	PV	66	Acute leukemia	23	Not available	*IDH1* c.394C>A p.Arg132Ser (1.3%)<br>*DDX41* c.1574G>A p.Arg525His (1.8%)<br>*JAK2* p.1849G>T p.Val617Phe (76.1%)<br>*ARID1A* c.6420dup p.Phe2141LeufsTer9 (0.6%)<br>*ASXL1* c.2766_2769dup p.Pro924Ter (0.4%)<br>*TET2* c.4045-1G>T (9.4%)<br>*TET2* c.5620G>T p.Glu1874Ter (3.2%)<br>*BRCA2* whole gene deletion	Failed remission (11 months)
81	M	64	PV	69	Acute leukemia	5	*JAK2* c.1849G>T p.Val617Phe (39.9%)<br>*SF3B1* c.1998G>T p.Lys666Asn (44.1%)	*CEBPA* c.68dup p.His24AlafsTer84 (86.0%)<br>*SF3B1* c.1998G>T p.Lys666Asn (49.2%)	Remission, Engraftment
82	M	78	PV	84	Acute leukemia	6	*JAK2* V617F PCR positive	*JAK2* c.1849G>T p.Val617Phe (90.9%)<br>*TP53* c.464C>A p.Thr155Asn (80.9%)	Death (1 month)

								TP53 whole gene deletion<br>*NF1* whole gene deletion	
83	M	73	PV	80	Acute leukemia	7	*JAK2* exon 12 direct sequencing, c.1614_1616delinsATT, p.His538_Lys539delinsGlnLeu	*JAK2* c.1614_1616delinsATT p.His538_Lys539delinsGlnLeu (70.1%)<br>*TP53* c.214_215insG p.Pro72ArgfsTer77 (62.3%)<br>*TP53* c.524G>A p.Arg175His (36.7%)	Death (4 days)
85	M	63	PV	66	Acute leukemia	3	*JAK2* V617F PCR positive	*JAK2* c.1849G>T p.Val617Phe (63.7%)	Remission, Engraftment
89	F	48	PV	68	Acute leukemia	20	*JAK2* V617F PCR positive	*JAK2* c.1849G>T p.Val617Phe (89.4%)<br>*TP53* c.218del p.Val73GlyfsTer50 (24.7%)<br>*ASXL1* c.2066C>G p.Ser689Ter (18.6%)	Death (2 months)
48	M	60	PMF	62	Acute leukemia	2	*JAK2* c.1849G>T p.Val617Phe (2.0%)<br>*SRSF2* c.284C>A p.Pro95His (45.3%)	*RUNX1::MECOM* rearrangement<br>*SRSF2* c.284C>A p.Pro95His (46.5%)<br>*KRAS* c.173C>T p.Thr58Ile (0.7%)	Death (9 months)
84	M	23	PMF	25	Acute leukemia	2	*JAK2* c.1849G>T p.Val617Phe (12.4%)<br>*U2AF1* c.101C>T p.Ser34Phe (34.5%)	No mutation detected	Death (7 months)

**JAK2* p.V617F mutation analysis was conducted using qualitative real-time PCR, and *JAK2* exon 12 was analyzed by direct sequencing, while all other mutation results were derived from next-generation sequencing.

Abbreviations: ET, essential thrombocythemia; PV, polycythemia vera; PMF, primary myelofibrosis; MPN, myeloproliferative neoplasm; VAF, variant allele frequency

Supplementary Table S4. Demographics in patients with myeloproliferative neoplasm that progressed to myelofibrosis

Patient No.	Sex	Age at MPN diagnosis	MPN	Age at progression to myelofibrosis	sMF	Time to progression to myelofibrosis (years)	Molecular result at MPN diagnosis*	Molecular result at progression to myelofibrosis (VAF)*	Status after MF diagnosis
15	F	57	ET	83	Post-ET MF	26	Not available	*DNMT3A* c.2645G>A p.Arg882His (47.9%)<br>*JAK2* c.1849G>T p.Val617Phe (86.1%)	Not progressed (5 years)
133	F	40	ET	60	Post-ET MF	20	Not available	*JAK2* c.1849G>T p.Val617Phe (65.2%)<br>*ASXL1* c.2077C>T p.Arg693Ter (21.3%)	Not progressed (4 months)
39	F	40	ET	54	Post-ET MF	14	Not available	*JAK2* c.1849G>T p.Val617Phe (91.6%)	Not progressed (3 years)
134	F	49	ET	65	Post-ET MF	16	*JAK2* V617F PCR negative<br>*JAK2* exon 12 direct sequencing, no mutation	*CALR* c.1099_1150del p.Leu367ThrfsTer46 (5.6%)<br>*TP53* c.772G>A p.Glu258Lys (3.3%)	Not progressed (6 months)
65	F	37	ET	47	Post-ET MF	10	*JAK2* V617F PCR positive	*JAK2* c.1849G>T p.Val617Phe (87.9%)	Not progressed (1 year)
68	F	41	ET	49	Post-ET MF	8	*JAK2* V617F PCR negative<br>*CALR* sequencing, c.1099_1150del p.Leu367ThrfsTer46	*CALR* c.1099_1150del p.Leu367ThrfsTer46 (28.2%)	Not progressed (2 years)
22	F	64	ET	65	Post-ET MF	1	*JAK2* V617F PCR negative<br>*JAK2* exon 12 no mutation	*CALR* c.1122_1125del p.Lys374AsnfsTer55 (23.5%)	Not progressed (4 years)

20	F	52	PV	79	Post-PV MF	27	Not available	*JAK2* c.1849G>T p.Val617Phe (93.4%)	Not progressed (4 years)
41	M	53	PV	67	Post-PV MF	14	*JAK2* V617F PCR positive	*JAK2* c.1849G>T p.Val617Phe (80.4%)	Not progressed (3 years)
27	M	55	PV	64	Post-PV MF	9	*JAK2* V617F PCR positive	*JAK2* c.1849G>T p.Val617Phe (79.2%)	Not progressed (4 years)
28	F	57	PV	65	Post-PV MF	8	*JAK2* V617F PCR positive	*JAK2* c.1849G>T p.Val617Phe (94.7%)	Not progressed (4 years)
57	F	41	PV	50	Post-PV MF	9	*JAK2* V617F PCR positive	*JAK2* c.1849G>T p.Val617Phe (89.1%) *NFE2* c.734dup p.Leu245PhefsTer5 (35.3%)	Not progressed (2 years)
60	M	67	PV	77	Post-PV MF	10	*JAK2* V617F PCR positive	*JAK2* c.1849G>T p.Val617Phe (87.7%)	Not progressed (2 years)
21	F	53	PV	59	Post-PV MF	6	*JAK2* V617F PCR positive	*JAK2* c.1849G>T p.Val617Phe (65.4%) *TET2* c.2634dup p.Leu879SerfsTer22 (35.7%)	Not progressed (4 years)
49	F	42	PV	47	Post-PV MF	5	*JAK2* V617F PCR positive	*JAK2* c.1849G>T p.Val617Phe (85.7%)	Not progressed (2 years)

**JAK2* V617F mutation analysis was conducted using qualitative real-time PCR, while *JAK2* exon 12 and *CALR* mutations were analyzed by direct sequencing. The results for all other mutations were derived from next-generation sequencing.

Abbreviations: ET, essential thrombocythemia; PV, polycythemia vera; PMF, primary myelofibrosis; MPN, myeloproliferative neoplasm; sMF, secondary myelofibrosis; MF, myelofibrosis; VAF, variant allele frequency
